# Microbial Diversity of Upland Rice Roots and Their Influence on Rice Growth and Drought Tolerance

**DOI:** 10.3390/microorganisms8091329

**Published:** 2020-08-31

**Authors:** Zhiqiang Pang, Ying Zhao, Peng Xu, Diqiu Yu

**Affiliations:** 1 CAS Key Laboratory of Tropical Plant Resources and Sustainable Use, Xishuangbanna Tropical Botanical Garden, Chinese Academy of Sciences, Kunming 650223, China; pangzhiqiang@xtbg.ac.cn (Z.P.); zhaoying@xtbg.ac.cn (Y.Z.); 2 Center of Economic Botany, Core Botanical Gardens, Chinese Academy of Sciences, Menglun, Mengla, Yunnan 666303, China; 3 College of Life Sciences, University of Chinese Academy of Sciences, Beijing 100049, China; 4 Center for Integrative Conservation, Xishuangbanna Tropical Botanical Garden, Chinese Academy of Sciences, Mengla, Yunnan 666303, China; 5 The Innovative Academy of Seed Design, Chinese Academy of Sciences, Menglun, Mengla, Yunnan 666303, China; 6 State Key Laboratory for Conservation and Utilization of Bio-Resources in Yunnan, Yunnan University, Kunming 650091, China

**Keywords:** rice ecotypes, PGPR, rhizosphere, endophytic fungi, antioxidant

## Abstract

Among abiotic stresses, drought is one of the most important factors limiting plant growth. To increase their drought tolerance and survival, most plants interact directly with a variety of microbes. Upland rice (*Oryza sativa* L.) is a rice ecotype that differs from irrigated ecotype rice; it is adapted to both drought-stress and aerobic conditions. However, its root microbial resources have not been explored. We isolated bacteria and fungi from roots of upland rice in Xishuangbanna, China. Four hundred sixty-two endophytic and rhizospheric isolates (337 bacteria and 125 fungi) were distributed. They were distributed among 43 genera on the basis of 16S rRNA and internal transcribed spacer (ITS) gene sequence analysis. Notably, these root microbes differed from irrigated rice root microbes in irrigated environments; for example, members of the Firmicutes phylum were enriched (by 28.54%) in the roots of the upland plants. The plant growth-promoting (PGP) potential of 217 isolates was investigated in vitro. The PGP ability of 17 endophytic and 10 rhizospheric isolates from upland rice roots was evaluated under well-irrigated and drought-stress conditions, and 9 fungal strains increased rice seedling shoot length, shoot and root fresh weight (FW), antioxidant capability, and proline (Pro) and soluble sugar contents. Our work suggests that fungi from upland rice roots can increase plant growth under irrigated and drought-stress conditions and can serve as effective microbial resources for sustainable agricultural production in arid regions.

## 1. Introduction

Drought is one of the major constraints of food security and plant productivity worldwide. Drought has a negative impact on the growth, development and agronomic yield of crops [1] and can degrade land [2]. The Intergovernmental Panel on Climate Change (IPCC) reported that the frequency of drought will increase by the end of this century because of climate change [3]. Drought stress has several effects on plants; for example, ion stress and osmotic stress cause oxidative stress in plants, resulting in the production of reactive oxygen species (ROS), which are harmful to plants [4]. Traditional research on promoting drought tolerance in plants mainly involves molecular marker-assisted breeding and genetic engineering. The basic strategy is to introduce functional genes that are directly involved in the plant response to drought through molecular breeding [5]. Unfortunately, these methods are highly labor intensive and technical and thus are currently difficult to apply in agricultural practice [6]. In addition, public awareness of genetically modified (GM) foods and safety supervision of GM techniques, as well as other techniques, may be needed.

Another strategy for growing plants under drought conditions is the use of plant growth-promoting rhizobacteria (root), bacteria and fungi (PGPR, PGPB, and PGPF, respectively). A great variety of microbes are found to inhabit plant surfaces and different plant tissues and organs, including roots, rhizosphere, phyllosphere, stems, leaves, flowers, and seeds [7,8,9]. Root-associated microbes can improve host plant growth (such as N fixation [10], phosphate solubilization [11] and iron chelation), suppress pathogens, and mobilize some micronutrients, and they offer the potential to increase crop plant resilience to future drought [12]. Microbial-based plant biotechnology has been proven to be more effective than plant breeding and genetic improvement methods [13]. A recent study has shown that endophytes improve plant tolerance against abiotic stresses such as salinity, drought and cold [6,14,15,16,17,18,19]. PGPB and fungi can alleviate abiotic stresses of their host plants via a variety of mechanisms [20,21,22]. Lowering ethylene levels by 1-aminocyclopropane-1-carboxylate (ACC) deaminase is considered one of the major mechanisms employed by PGPB to promote plant growth under stress conditions [23,24]. It has been verified that certain bacteria are able to produce ACC deaminase to convert the ethylene precursor ACC into α-ketobutyrate and ammonia [25]. By degrading ACC within roots or in exudates [26], root-interacting bacteria can reduce ethylene levels in plant roots [27,28], stimulate root growth and improve plant drought tolerance [29,30]. The production of exopolysaccharides (EPS) also plays an important role in supporting plant growth under drought-stress conditions [14,16,31]. Studies have shown that EPS promote improved drought tolerance in wheat [14], corn [32], rice [11,15], and pepper [31]. Another group of microbes, mycorrhizal fungi, promote the metabolism and development of plants by facilitating plant water uptake via their extensive hyphae [33,34]. Furthermore, PGPB and PGPF (include mycorrhizal fungi) can increase plant stress tolerance by inducing the accumulation of osmolytes [35] and antioxidants [24,36], regulating stress response genes [37,38], increasing mineral nutrition and photosynthesis, and altering root morphology [39].

Rice (*Oryza sativa* L.) exists as two ecotypes, upland and irrigated ecotypes, which are adapted to rainfed upland conditions and well-watered conditions, respectively [40,41]. However, previous studies on the rice microbiome or PGPR have focused mainly on irrigated rice [11,42,43,44,45,46,47,48]. Recent studies have proven that there may be great differences between root microbes of irrigated rice and drought-stressed rice [10,49,50]. Therefore, it is necessary to isolate root microbes of upland rice (that growing under persistent, relatively dry conditions compared with irrigated rice), which could provide microbial resources for improving the drought tolerance of rice and other upland crop species. In this work, a wide range of bacteria and fungi were isolated from the roots (rhizospheric and endophytic types) of upland rice in Xishuangbanna (Southwest China). We then determined their plant growth-promoting (PGP) ability in vitro. Furthermore, we investigated their effects on rice plant growth under well-irrigated and drought-stress conditions. These microbes could offer high potential for increasing the resiliency of crop plant production systems to drought.

## 2. Materials and Methods

### 2.1. Upland Rice Samples

The roots of upland rice (*O. sativa*) were collected at the molecular breeding station at Xishuangbanna Tropical Botanical Garden (XTBG), Chinese Academy of Sciences (Latitude 21.16° N; longitude 101.04° E, Menglun, Yunnan Province, Southwest China). The station has international upland rice and local upland rice varieties grown in China (different genotypes and *Indica* and *Japonica* varieties are also present), such as Luying 46, Azucena, PHB-XTBG, PHB1, Mengwanggu and Xiaojingu. The upland fields in the XTBG Breeding Station have been used for irrigated rice and upland rice breeding for the past 5 years. After their surface was sterilized, the rice seeds were sown in the upland fields. In October 2019, we collected the above-mentioned upland varieties healthy root samples, placed them in ice boxes and then brought the samples back to the laboratory immediately. The rice root samples were then washed carefully under tap water to remove any surface soil.

### 2.2. Isolation of Bacteria and Fungi of the Roots

#### 2.2.1. Isolation of Rhizosphere Bacteria and Fungi

Fungi and bacteria associated with rhizosphere soils (within a 1 mm vicinity of the roots) were isolated using the traditional serial dilution technique [20,51]. Afterward, 1 g of each rhizosphere soil sample was added to 99 mL of ddH_2_O, and a 10-fold serial dilution was made from the suspension (10^−3^ to 10^−6^). Dilutions of 10^−4^ and 10^−5^ were used to isolate fungi, and 10^−5^ and 10^−6^ were used to isolate bacteria. One hundred microliters of each diluted suspension was spread evenly onto potato dextrose agar (PDA, Hopebio, Qingdao), malt extract agar (MEA, Huankai Microbiol, Guangzhou) and Czapek-Dox media (CDM, Huankai Microbiol, Guangzhou) (to isolate the fungi) or onto tryptic soy agar (TSA, Hopebio, Qingdao) (to isolate the bacteria). We added 0.02 g L^−1^ tetracycline hydrochloride and 0.05 g L^−1^ streptomycin sulfate to the PDA to eliminate bacterial contamination. The PDA plates were incubated at 28 °C in darkness for 3 days, and the TSA plates were incubated for 1–2 days until colonies appeared. The colonies were removed and transferred to fresh PDA or TSA plates for purification.

#### 2.2.2. Isolation of Endophytes

Surface sterilization of the upland rice roots was performed according to a previously described procedure [20,52,53]. The root samples were washed under running tap water to remove bulk soil and other loosely attached debris. The clean roots were then cut into 3–5 cm pieces and surface sterilized using 1% NaClO (10 min), 75% alcohol (3 min), and 95% alcohol (30 s, 3 times). Finally, the sections were rinsed three to five times with sterile distilled water. The endophytic bacteria were isolated using the fragmentation technique [53]. With respect to fungi, dried root samples were placed onto sterile filter paper, cut into 8–10 mm segments and then placed onto plates of PDA supplemented with antibiotics as mentioned above. The PDA plates were incubated for 3–5 days at 28 °C in the dark, after which emerging fungal hyphae growing from the tissue fragments were transferred to PDA for purification [20]. Pure cultures were subsequently stored at −20 °C and −80 °C in glycerol stock solution until processed.

### 2.3. DNA Extraction, Genotyping and Identification of Bacteria and Fungi

The genomic DNA from pure bacterial cultures grown overnight in tryptic soy broth (TSB) at 28 °C was extracted using an *EasyPure^®^* Bacteria Genomic DNA Kit (TransGen, Beijing) according to the manufacturer’s instructions. The fungal mycelia were scraped from the PDA plates and transferred to a sterile 1.5 mL tube. Fungal DNA was extracted using a Fungi Genomic DNA Extraction Kit (Solarbio, Beijing) according to the manufacturer’s instructions. The 16S rRNA gene was amplified by PCR using the universal primers 799F (5′-AACMGGATTAGATACCCKG-3′) and 1193R (5′-ACGTCATCCCCACCTTCC-3′) [45]. The internal transcribed spacer (ITS) primers used were ITS1F (5′-CTTGGTCATTTAGAGGAAGTAA-3′) and ITS4 (5′-TCCTCCGCTTATTGATATGC-3′) [20,54]. The final volume of the PCR solution was 50 µL, which consisted of 2 µL of template DNA (20–50 ng), 0.5 µL of each forward and reverse primer (50 µM, 10P), 25 µL of 2 × Taq Master Mix (Tsingke, Beijing, China), and 22 µL of sterile deionized water.

PCR was performed in a thermal cycler under different conditions depending on the primers used. For 16S rRNA, after an initial denaturation step at 98 °C for 30 s, the targeted region was amplified by 25 cycles of 98 °C for 10 s, 55 °C for 15 s and 72 °C for 60 s, followed by a final elongation step of 5 min at 72 °C. For ITSs, there was a predenaturation step at 94 °C for 4 min; 35 cycles of denaturation at 94 °C for 40 s, annealing at 55 °C for 50 s and elongation at 72 °C for 1 min; and a final extension at 72 °C for 10 min. All purified PCR products were sent to Beijing Tsingke Biotech, Co., Ltd. (Kunming, China) and sequenced with the above primer pairs. The bacterial 16S rDNA and fungal ITS nucleotide sequences were aligned with known sequences in the NCBI (http://blast.ncbi.nlm.nih.gov), Ribosomal Database Project (https://rdp.cme.msu.edu/, RDP) and UNITE (https://unite.ut.ee/analysis.php) databases via BLASTn for initial identification, the results of which are listed in Table S1. All the sequences from this study have been deposited in the National Genomics Data Center BioProject (Biological Project Library) and Genome Sequence Archive (GSA) Read Archive under Accession No. PRJCA003044 (the sequence dataset is available at https://bigd.big.ac.cn/gsub/).

### 2.4. In Vitro Characterization of Isolates for PGP Traits and Drought Tolerance

The PGP traits, including N fixation ability, phosphate solubilization ability, ACC deaminase activity, siderophore production and drought tolerance, were determined by following standard procedures. The N fixation ability was determined by observing growth on Ashby media [53], and the *nifH* gene, which represents N fixation ability, was also amplified using the primers PolF (5′-TGCGAYCCSAARGCBGACTC-3′) and PolR (5′-ATSGCCATCATYTCRCCGGA-3′) [6,55]. To determine the phosphate solubilization ability, bacterial and fungal cultures were transferred to organophosphorus media and inorganic phosphorus media (Hopebio, Qingdao) and incubated at 28 °C for 48–72 h. The phosphate solubilization ability was checked according to the halos around the colonies [56,57].

Siderophore production by bacterial and fungal isolates was determined via the universal assay involving the use of CAS-blue plates as described by Ji et al. [56,58]. The color of the CAS-blue agar plates incubated at 30 °C for 48 h changed to orange, indicating the presence of siderophores after chelation of bound iron. The ACC deaminase activity of the bacterial isolates was screened based on the ability to use ACC as a sole N source and on the *acdS* gene. Isolates were spot inoculated on DF salt minimal agar media [6,59] supplemented with 3 mM ACC instead of (NH_4_)_2_SO_4_ as a N source, after which the presence of the ACC deaminase gene *acdS* was determined [60,61,62]. The *acdS* gene was amplified by PCR using the degenerate primers acdSf3 (5′-ATCGGCGGCATCCAGWSNAAYCANAC-3′) and acdSr4 (5′-GGCACGCCGCCCARRTGNRCRTA-3′) [29,63,64].

Bacterial and fungal growth were measured under increasing polyethylene glycol (PEG) levels in the culture media [6,65]. Sterile TSB consisting of 0%, 5%, 10%, 15%, and 25% PEG 6000 was developed and inoculated with bacteria in a 50 mL tube at 28 °C in a shaking incubator at 150 rpm for 72 h. PDA culture media consisting of only 10% PEG was used, and the appropriate amounts of plant gelling agents were added to solidify the culture media. Fungi were transferred to the media and cultured at 28 °C for 3–5 days.

Bacterial isolates were further assayed for the production of EPS according to the protocol suggested by Ali et al. [66], with slight modifications. For EPS extraction, the cultures were centrifuged at 15,000 rpm for 25 min at 4 °C, after which the supernatant was collected.

### 2.5. Rice (O. sativa) Growth Promotion Assays

The strains exhibiting both good drought tolerance and multiple plant growth promoting traits were selected from the dominant in vitro groups and were further evaluated their effect on rice seedling growth. To prepare the spore suspensions, fungi growing on PDA were sub-cultured in 100 mL of liquid potato dextrose broth (PDB) at 27 °C with shaking at 160 rpm for 7 days. One milliliter of pure bacterial culture (10^8^ CFU mL^−1^) grown in TSB for 48 h at 28 °C was applied to the appropriate pots at sowing just below the seedlings. Five days later, bacterial and fungal cultures (1 mL of culture and 50 mL of distilled water) were applied around the seedlings [67].

The seeds of rice (irrigated rice Zhongkexilu4, an *O. sativa indica* from the XTBG Breeding Station) were sterilized with 75% ethanol for 3 min and 1% NaClO for 10 min. The sterilized seeds were rinsed with distilled water and then aseptically planted into homogenized bacterial and fungal liquid media in Petri dishes for germination at 28 °C for 12 h. Following pre-germination, the seedlings were transferred to sterile pots (8 cm diameter, 18 cm height; 2 seedlings pot^−1^) containing 300 g of soil media, which consisted of soil from their natural habitat (pH 5.32 ± 0.15, Organic matter 11.83 ± 0.08 g kg^−1^, Available N 76.96 ± 7.33 mg kg^−1^, Available P 13.04 ± 0.39 mg kg^−1^, Available K 63.33 ± 1.18 mg kg^−1^, Fe 205.67 ± 4.43 mg kg^−1^), humus (with an amount of organic material) and quartz sand (v:v:v), after which the pots were autoclaved three times for 120 min at 121 °C.

The experiment involved a completely randomized design, with an inoculation treatment (non-inoculated controls (CKs) are designated as CK-TSB (Bacteria group) and CK-PDB (Fungi group)) and a natural drought treatment as the variables for plant species. Each treatment was replicated in six experimental pots. All the pots were maintained in a growth chamber at 28 °C during the day and 22 °C during the night under a photoperiod of 14 h (6:00–20:00), and the mean relative humidity was 60%. After 20 days, the rice seedings were moved to the greenhouse of the breeding station and received natural drought (the drought group no watering).

### 2.6. Plant Harvest and Biochemical Analyses

At one week after planting and no watering, irrigation was resumed for 1 day, plant health was assessed and imaged [14], and the plants were harvested (three CK and drought replications per treatment). The fresh weight (FW), length of the roots and shoots and leaf chlorophyll contents were measured. Before harvest, several physiological values such as shoot length were measured (see below). Leaves from each treatment were collected and used for measurements of shoot proline (Pro), soluble sugar, malondialdehyde (MDA), superoxide dismutase (SOD), peroxidase (POD), catalase (CAT), and total chlorophyll contents, as described in a previous study [14]. Total chlorophyll a (Chla) and chlorophyll b (Chlb) contents were analyzed according to the methods in a previous study [16]. Fresh parietal lobe leaf material (0.1 g) was collected, frozen in liquid N and ground into powder with a mortar and pestle. The total pigments were extracted after the material was ground and then extracted with 2 mL of 90% acetone. The crude extracts were then centrifuged at 1500 *g* for 5 min. The supernatant was kept, and the pellet was discarded [68]. Quantification was performed via spectrophotometry at 663 nm and 645 nm. The amount of chlorophyll was calculated according to the following formulas: Chla (μg mL^−1^, FW) = 12.72A_663_ − 2.59A_645_; Chlb (μg mL^−1^, FW) = 20.31A_645_ − 4.91A_663_; and *C_Chl_* (μg mL^−1^, FW) = 20.29A_645_ + 8.05A_663_ [69].

### 2.7. Statistical Analyses

Analysis of variance (ANOVA) of the obtained numerical data was performed using SPSS 20 (IBM Corporation, USA) statistical software. The mean values of the treatments were separated using Duncan’s multiple range test at *p* ≤ 0.05. The results are expressed as the means ± SDs of the means [65].

## 3. Results

### 3.1. Diversity of Culturable Rhizosphere and Endophytic Bacteria and Fungi in Upland Rice Roots

**Figure 1:**
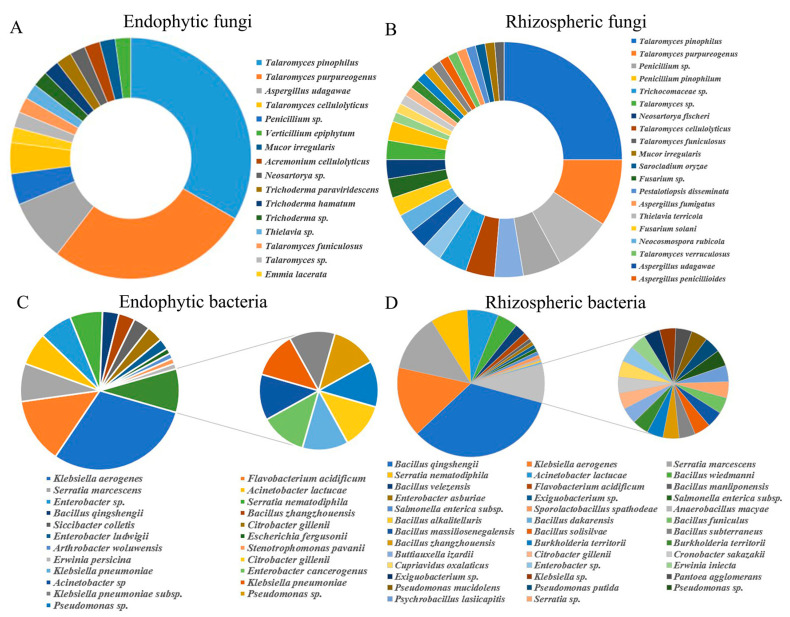
Taxonomic affiliations based on partial 16S rRNA and internal transcribed spacer (ITS) gene sequencing analysis: The relative abundance of cultivable bacteria and fungi inhabiting upland rhizosphere and endophyte. (**A**) Fungi isolated from endophyte, (**B**) Fungi isolated from rhizosphere, (**C**) Bacteria isolated from endophyte, (**D**) Bacteria isolated from rhizosphere.

Analysis of the root communities of upland rice resulted in the isolation of 337 bacteria (91 endophytes and 246 rhizosphere) and 125 fungi (76 endophytes and 49 rhizosphere). Based on their partial 16S rRNA and ITS gene sequences, 462 isolates provided good-quality sequences and were identified as diverse bacterial and fungal species via phylogenetic classification based on the NCBI, RDP and UNITE database information. Based on the BLAST results, culturable bacterial and fungal isolates were distributed among 62 bacterial species (24 endophytes and 38 rhizosphere microbes) and 47 fungal species (30 endophytes and 17 rhizosphere microbes) that belong to 43 genera (26 bacteria and 17 fungi), 31 families (19 bacteria and 12 fungi), 19 orders (10 bacteria and 9 fungi), 13 classes (8 bacteria and 5 fungi), and 8 phyla (5 bacteria and 3 fungi) (Figure 1 and Table S1). *Talaromyces pinophilus* was the most abundant species in the fungal community of the upland rice roots, representing 25% and 33% of the isolates of the endophytes and rhizosphere microbes, respectively (Figure 1A,B and Table S1.). *Bacillus qingshengii* was the most abundant (33.74%) species in the rhizosphere, and it was found exclusively in the rhizosphere. The relative abundance of *Klebsiella aerogenes* was 30% and 15.45% among the endophytes and rhizosphere microbes, respectively. *Acinetobacter lactucae* was present at similar proportions in different niches—7% and 6.91% among the endophytes and rhizosphere microbes, respectively. Members of the *Serratia* genus constituted 7% and 20% of the relative abundance of the endophytes and rhizosphere microbes, respectively. These isolates were members of *Klebsiella*, *Serratia*, *Pantoea*, *Enterobacter*, and so on, and the first to third were the predominant genera (32.97%, 14.29%, and 13.19%, respectively.) among the bacterial and fungal communities of the upland roots. There were 109 isolates of *Bacillus* (44.31%), which was the most dominant bacterial genus in the rhizosphere. Following the *Bacillus* isolates were those of *Serratia* (52 isolates, 21.14%), *Klebsiella* (39 isolates, 15.85%), and *Acinetobacter* (17 isolates, 6.91%). The diversity of the rhizosphere bacteria (19 genera) was higher than that of the endophytic bacteria (14 genera); however, the abundance of all genera except *Talaromyces* (43.42% and 67.35%) was relatively small in both the endophyte and rhizosphere fungal communities. *Penicillium* and *Thielavia* were more abundant within the endophyte community (13.16% and 11.84%, respectively) than within the rhizosphere community (4.08% and 2.04%, respectively).

### 3.2. Assessment of PGP Traits of Microbes In Vitro

A total of 217 isolates from the bacterial group (92 isolates) and fungal group (125 isolates) were tested in vitro for traits related to PGP activity. The most widespread activities within the selected bacterial isolates were phosphate solubilization, N fixation and drought tolerance, and none of the strains exhibited siderophore production (Table S2). Among the strains, most *Bacillus* species produced clear dissolution halos, and *Pantoea* species produced the largest halo (+++). Some endophytic fungi such as *Talaromyces* spp. and some rhizospheric bacteria such as *Serratia* spp. can dissolve organophosphorus and inorganic phosphorus. Most of the bacteria screened further showed drought tolerance potential (OD_600_ > 0.8) and contained the *acdS* gene. These results indicated that upland rice endophytes and rhizosphere microbes have high PGP potential and may be good candidate strains for use as biofertilizers for rice. Fourteen bacteria and 13 fungi with PGP ability were ultimately selected from the 217 isolates to inoculate rice seedlings (Table 1).

### 3.3. Effects of Fungi on Rice Seedling Growth

**Figure 2:**
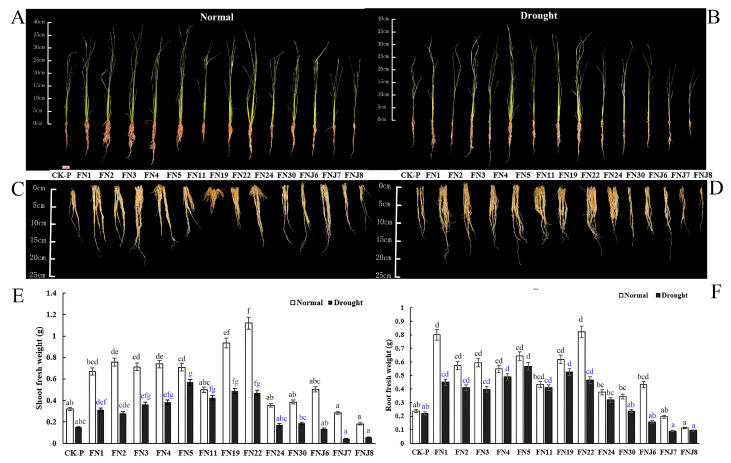
Differential fungal effects on rice growth and drought tolerance. (**A**–**D**) Representative images of rice shoot and root inoculated with endophyte and rhizosphere fungi compared to non-inoculated plants under well-irrigated and drought stress. (**A**) Rice images under well-irrigated. (**B**) Rice images under drought stress. (**C**) Rice root images under well-irrigated. (**D**) Rice root images under drought stress. (**E**) Shoot fresh weight. (**F**) Root fresh weight. *N* = 14. Abbreviations shown in Table 1. Different lowercase letters indicate significant differences of shoot or root fresh weight among treatments (ANOVA analysis, Means followed by the different letter are significantly different, *p*  = 0.05).

After performing media screening and in vitro tests, we selected 14 bacteria (7 endophytes and 7 rhizosphere microbes) and 13 fungi (10 endophytes and 3 rhizosphere microbes) with which to inoculate rice seedlings (Table 1). We further studied the effects of different endophytic and rhizosphere fungi on the rice growth and drought tolerance (Figure S1). Upon inoculation, fungi promoted rice growth, as indicated by the significantly greater plant shoot and root FW compared to that of the CK-P (CK) under both non-stressed conditions and drought-stress conditions (Figure 2). After seven days of water deprivation, the non-inoculated CK rice became wilted and desiccated, whereas the inoculated plants were not severely affected, with only slight curling of the leaf tips (Figure 2A–D). Compared to that of the non-inoculated stressed CK rice, the shoot and root FW of the inoculated rice increased significantly (Figure 2A–F). Under non-stressed conditions, fungi FN22 (*Chaetomium pilosum*) and FN19 (*Aspergillus aureolus*) increased the FW of aerial parts of the rice seedlings (by 364.69% and 280.15%, respectively); in addition, fungi FN22 (*Chaetomium pilosum*) and FN1 (*Talaromyces pinophilus*) also significantly increased the FW of rice roots (247.33% and 238.47%, respectively). Under drought stress, FN22 (*Chaetomium pilosum*) increased the shoot FW of rice (216.05%), and FN5 (*Talaromyces purpureogenus*) increased the shoot and root FW to the greatest degree (by 280.21% and 158.75%, respectively). Similarly, in response to FN19 (*Aspergillus aureolus*) inoculation, the FW of rice roots increased significantly (138.85%). Other selected endophytic and rhizosphere fungi also presented obvious growth-promoting effects. However, inoculation FNJ7 (*Trichocomaceae* sp.) and FNJ8 (*Hypocreales* sp.) reduced plant growth under non-stressed conditions (−16.25% and −62.58% of shoot FW, respectively; −17.39% and −51.70% of root FW, respectively) and under drought-stress conditions (−70.66% and −61.51% of shoot FW, respectively; −59.05% and −56.93% of root FW, respectively). The above results indicated that inoculating upland rice with endophytic or rhizosphere fungi has a positive impact on rice growth under both non-stressed and drought-stress conditions, suggesting that these isolates play a dual role in increasing rice plant growth and drought stress tolerance (Figure S1).

**Figure 3:**
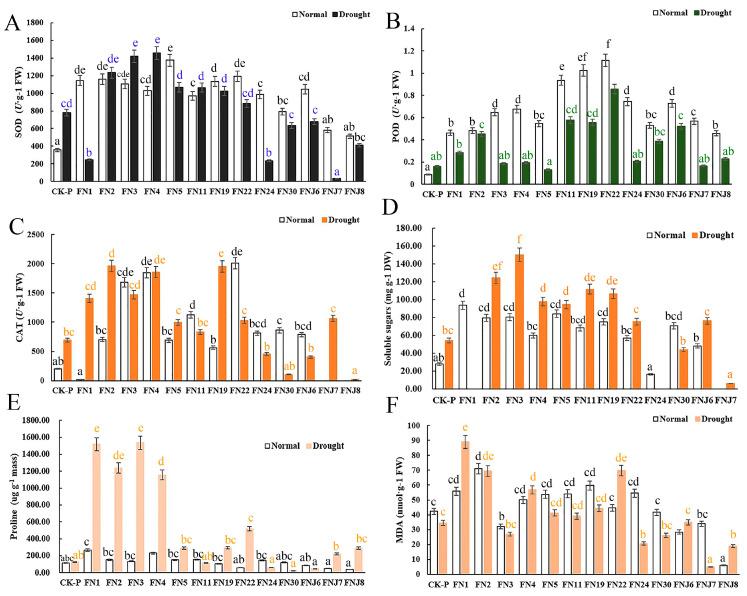
Differential fungal effects on rice physiological indices. (**A**–**F**) Respectively represent the contents of superoxide dismutase (SOD), peroxidase (POD), catalase (CAT), proline, plant soluble sugars, and malondialdehyde (MDA) in the rice. *N* = 14. Different lowercase letters indicate significant differences of shoot or root fresh weight among treatments (ANOVA analysis, Means followed by the different letter are significantly different, *p*  =  0.05).

To evaluate how fungi promote plant growth and alleviate drought stress, physiological parameters, including plant soluble sugar, Pro, MDA, and leaf chlorophyll contents, and activities of antioxidant enzymes, such as superoxide dismutase (SOD), peroxidase (POD), and catalase (CAT), were measured (Figure 3). Under non-stressed conditions, rice inoculated with fungi presented increased CAT, POD, and SOD activities as well as increased soluble sugar, Pro and chlorophyll contents. Moreover, the MDA content was higher in these rice plants compared with the non-inoculated CK rice plants (Figure 3F). After the plants were exposed to drought stress, the Pro and soluble sugar contents and the SOD, CAT, and POD activities increased significantly in the rice inoculated with fungi compared to the non-inoculated CK rice. The antioxidant enzyme activity and plant soluble sugar contents were greater in the drought-stressed plants inoculated with fungi than in the non-inoculated CK plants. In addition, compared with those in the CK plants, the concentrations of MDA in the inoculated plants increased. Like the phenotypic results, the rhizosphere-inhabiting FNJ7 (*Trichocomaceae* sp.) and FNJ8 (*Hypocreales* sp.) also exerted no growth-promoting effects on the physiological parameters, and the antioxidant enzyme activities of the plants inoculated with these fungi were significant lower than those of CK. Taken together, these results suggested that the majority of here tested fungi may be involved in antioxidant metabolic pathways and may regulate the activity of antioxidant enzymes.

### 3.4. Effects of Upland Rice Root Bacteria on Plant Growth

**Figure 4:**
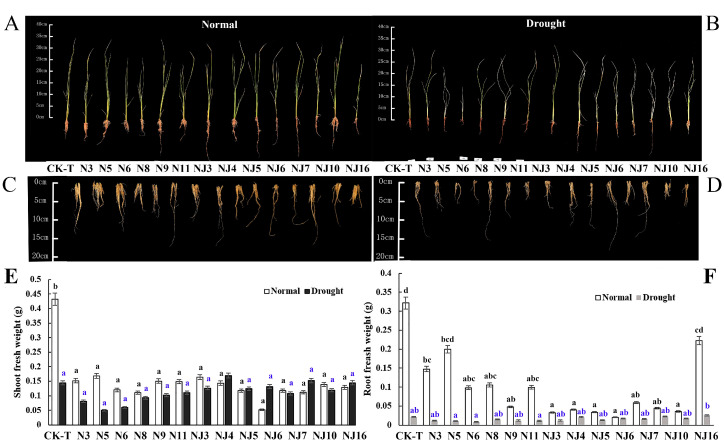
Differential bacterial effects on rice growth and drought tolerance. (**A**–**D**) Representative images of rice shoot and root inoculated with endophyte and rhizosphere bacteria compared to non-inoculated plants under well-irrigated and drought stress. (**A**) Rice images under well-irrigated. (**B**) Rice images under drought stress. (**C**) Rice root images under well-irrigated. (**D**) Rice root images under drought stress. (**E**) Shoot fresh weight. (**F**) Root fresh weight. *N* = 15. Different lowercase letters indicate significant differences of shoot or root fresh weight among treatments (ANOVA analysis, Means followed by the same letter are not significantly different, *p*  =  0.05).

Several bacterial strains (7 endophytes and 7 rhizosphere microbes (Table 1)), were inoculated and tested for their potential as PGPB in rice at the seeding stage. The results clearly showed that these 14 bacterial strains displayed variable effects on the chlorophyll content and the shoot and root length of rice (Figure S2). However, it was found that, under the pressure of natural drought, the bacteria strains negatively affected the growth of the rice seedlings (Figure 4). The negative effects on the shoots (−69.20% under no stress and −73.59% under drought stress) were more severe than were those on the roots (−21.90% under no stress and −28.52% under drought stress). With respect to plant FW, rhizosphere strain NJ5 (*Serratia* sp.) had the most obvious negative effects on the rice seedlings under non-stress conditions, decreasing the shoots and roots by −87.71% and −193.60%, respectively. Similarly, endophyte N5 (*Pantoea ananatis*) obviously hindered rice growth under drought stress, which was manifested by negative effects on the FW of both of shoots and roots (−58.13% and −61.41%, respectively). Phenotypic results of the bacterial group showed that the selected bacterial strains had no obvious PGP effects and caused the rice seedlings to wither and die; thus, with the exception of the chlorophyll content, no other biochemical indexes were measured (Figure S2).

## 4. Discussion

This study adopted a culture-centric approach to identify the endophyte and rhizosphere fungal and bacterial communities that colonize upland rice roots. To better understand the microbial community behavior in relation to the upland ecotype, we selected different genotypes of upland rice (including both international varieties and local Chinese varieties) cultivated in Xishuangbanna, Southwest China. Several studies have focused on different varieties present within irrigated rice root microbial communities; members of several bacterial groups, such as *Flavobacterium*, *Microbacterium*, *Pantoea*, *Serratia*, *Klebsiella,* and *Kosakonia*, and fungal groups, such as those isolated from most rice roots cultivars, have been identified [70,71]. Previous studies have indicated that the bacterial community within the root microbiome largely comprises Proteobacteria, Actinobacteria and Chloroflexi phyla [72]. Not surprisingly, the bacterial community composition in our study distinctly differs from that in the root bacteria of other irrigated rice ecotypes based on select reviews [71,72]. Notably, the Firmicutes phylum in the root is markedly enriched compared with that in the rhizosphere of irrigated rice. This could be in part due to the aerobic and drought adaptation characteristics of the upland rice roots; the environment may favor the growth and function of multiple monoderm (or gram positive) anaerobic bacteria, for instance, those of the Firmicutes phylum, which are more tolerant to desiccation than are diderms, due to the thicker cell walls of the former and the ability of some monoderms to sporulate [73,74]. The observation that monoderm enrichment is greatest in the upland root-associated fraction suggests that the enrichment described here is driven at least in part by an interaction within the upland rice and that increased monoderm abundance may promote increased drought tolerance of the upland rice. Like the bacterial community composition, the fungal community composition in the upland rice roots may differ from that in the rhizosphere of irrigated rice, particularly with no enrichment of members of the Chytridiomycota phylum, which are considered aquatic fungi and are ubiquitous in aquatic environments and habitats with high moisture (such as well-irrigated paddies) [72].

Given that drought has become one of the most common environmental abiotic stresses that reduces crop yield and productivity worldwide, drought-tolerant varieties cultivated through conventional breeding techniques and genetic engineering are widely used to mitigate the negative effects of drought stress on crop growth and yield [14,75,76]. However, most of these efforts are time consuming and expensive. In addition, public awareness and supervision of techniques involving things such as genetically modified organisms (GMOs) may be needed. Under such circumstances, plant-related microbes that can improve the drought tolerance of crops have attracted increased amounts of attention [33,49,77,78,79]. Scientists have isolated rhizospheric and endophytic bacteria and fungi from arid regions or from xerophytes, and these microbes have been shown to improve the growth of rice, sorghum, corn, cabbage, millet, and wheat under drought-stress conditions [14,80,81,82,83,84]. There is abundant evidence that root-associated microbes can help sustain plant growth under drought conditions [12,18]. However, no systematic study on the identity and PGP potential of upland rice root-associated microbes as well as their effects on drought tolerance exists. The PGP potential and drought tolerance trait assays showed that the highest numbers of positive isolates were observed among *Serratia*, *Pantoea*, *Flavobacterium*, *Pseudomonas*, *Klebsiella*, *Bacillus*, *Talaromyces*, *Trichoderma*, and *Penicillium* genera (Table S2). Microbes affiliated with these taxa, such as *Bacillus thuringiensis* and *Piriformospora indica*, have been widely reported to be PGPB and PGP fungi present in the root endosphere and rhizosphere for a range of xerophytes and crop plants [65].

Physiological parameters such as antioxidant enzyme (POD, SOD, CAT) activity, Pro content, MDA content and plant soluble sugar content are closely involved in the drought response [24,36]. ROS, whose production is a typical plant defense response, are usually strongly associated with abiotic stress. ROS are produced mainly by NADPH oxidase and are eliminated by antioxidant enzymes, such as SOD, CAT and POD. ROS are also essential components for the successful survival of colonizing endophytes during the plant oxidative burst [4,85]. In the present study, we identified a total of 10 endophytic (*Talaromyces pinophilus*, *Talaromyces purpureogenus*, *Penicillium* sp., *Talaromyces cellulolyticus*, *Talaromyces purpureogenus*, *Talaromyces flavus*, *Aspergillus aureolus*, *Chaetomium pilosum*, *Penicillium limosum*, and *Talaromyces purpureogenus*) and 3 rhizospheric fungus (*Talaromyces purpureogenus*, *Trichocomaceae* sp., and *Hypocreales* sp.) of upland rice that displayed obvious growth-promoting effects on rice seedlings under both irrigated and drought-stress conditions. Our results showed that the endophytic fungi of the upland rice roots may be associated with tolerance to osmotic stress and the ability to detoxify ROS in rice seedlings. In our experiment, the SOD, POD, and CAT activities in most rice seedlings inoculated with fungi increased. These fungal inoculants may be involved in antioxidant metabolic pathways and the regulation of antioxidant enzyme activity. In plants, total soluble sugar and Pro contents are critical biochemical markers of abiotic tolerance [86,87]. Soluble sugars play a role in drought tolerance by maintaining osmotic turgor. It has been reported that soluble sugar concentrations in plant leaves increase sharply under drought stress [14,86]. Similar to that in previous studies, the inoculants decreased the antioxidant activities and increased the production of Pro, free amino acids, and sugars in the plants [88]. Similarly, drought-tolerant bacterial strains inoculated into corn showed potential increases in Pro and sugar contents [89,90]. As expected, the soluble sugar content increased significantly in the drought-stressed plants in the present study, and the presence of several fungi further increased this content, suggesting that fungi contribute to increased accumulation of sugars for better osmotic adjustment, thus alleviating drought-induced damage in rice. Similar results were found for wheat under drought stress [14]. However, inoculation with some fungal strains (*Talaromyces pinophilus*, *Talaromyces purpureogenus*, *Talaromyces cellulolyticus*, *Chaetomium pilosum*) showed the opposite effects, in which the MDA content increased under both water stress and non-stressed conditions. The main results are such that several fungi (*Penicillium* sp., *Talaromyces purpureogenus*, *Talaromyces flavus*, *Aspergillus aureolus*, *Penicillium limosum*, and *Talaromyces purpureogenus*) alleviated oxidative stress in rice under drought-stress conditions, as evidenced by the decrease in MDA content compared with that in the CK. These findings indicated that upland rice root-associated fungi may help repair the damage to membrane integrity and functionality caused by water stress [91]. In summary, the effectiveness of fungal group strains in promoting the growth of rice seedlings under irrigated and drought-stress conditions.

Notably, most bacteria have no growth-promoting effect on rice seedlings under drought-stress conditions (natural dry condition and under high-temperature stress of which the instantaneous maximum temperature is 46.7 °C, which is close to the most extreme heat of climates in tropical regions). Many bacterial strains with PGP potential have been screened in the laboratory. The production of EPS by some PGPB can ameliorate the water-holding capacity of the soil and promote the stability of soil aggregates, which is particularly relevant in arid environments [92]. Moreover, despite widespread claims of efficacy of inoculation with PGPB under laboratory conditions, many studies have been unable to attribute the beneficial effects to a specific trait and suggest that untapped mechanisms await discovery [93], and there is limited evidence of inoculation success and subsequent benefits for plant growth under drought stress [12]. Thus, understanding the mechanisms through which root microbes affect plant drought tolerance and their relevance and applicability under realistic upland field and drought conditions offers much potential for increasing the resiliency of crop production systems to drought [12]. Previous studies have shown that plants reduce their supply of carbon to bacteria more than fungi do under drought conditions [94]. Compared with the fungal community, the soil bacterial community has poor stress resistance but strong recovery ability. Fungi are the first consumers of plant carbon inputted into the rhizosphere [95,96]. As such, we speculate that bacteria are more sensitive to environmental stress. Under the combined pressure of high temperature and drought, bacteria have no growth-promoting effects on rice seedlings. In this case, whether the root system of rice seedlings produces an immune response to prevent bacteria from damaging it needs further verification.

A previous study showed that applying adaptations to aquatic conditions (paddy fields) to aerobic environments (rainfed drylands) might be the main characteristic associated with the domestication of upland rice ecotypes during long-term selection and domestication [40,97]. Perhaps upland ecotype rice varieties have adapted to aerobic environments throughout the long-term evolution and domestication processes. Specific enrichment of the microbiome is needed for adaptations of irrigated ecotypes to dry upland conditions. Therefore, it is necessary to investigate the composition of the root microbiome of upland ecotype rice that grows under rainfed dryland conditions and in paddy fields and compare it with that of irrigated ecotype rice to provide microbial-level evidence for drought tolerance and adaptability of upland ecotype rice. Microbial-based plant biotechnology has been proven to be more effective than plant breeding and genetic improvement techniques. Therefore, it is necessary to clarify the molecular mechanism underlying the plant root-microbiome interaction system under drought stress and its mode of application. In recent years, studying the root microbial communities in different niches of rice based on high-throughput sequencing has become popular. It is therefore necessary to explore the actual community structure of the root system by combining metagenomics, plant metabolomics and other technical methods.

## 5. Conclusions

Xerophyte and plant adaptations to drought-stress and aerobic conditions, such as the adaptations of upland ecotype rice and plant microbiomes, are still an overlooked source of microbial resources that are potentially useful for agricultural biotechnology applications. Currently, no reports concerning the root microbe communities associated with upland rice are available. Upland rice is highly adapted to low water availability and increased drought-stress conditions. Their survival could be partly related to a significant reservoir of beneficial microbes associated with their roots. This is the first study to systematically analyze the diversity of culturable endophytic and rhizosphere bacteria and fungi in upland rice and demonstrate their functional role in plant drought tolerance. Notably, the root microbes of upland rice may differ from those of irrigated rice. The results of this study suggest that the roots of upland rice grown in tropical areas interact with a variety of endophytic and rhizosphere bacteria and fungi that display rice growth-promoting traits under both irrigated and drought-stress conditions. The fungi of upland rice roots could be a resource capable of being used to directly protect rice seedlings from drought stress. Our results suggest that some endophytic fungi of upland rice function to alleviate drought stress in rice by increasing antioxidant enzyme activity, Pro content, soluble sugar content and rice seedling growth. Furthermore, multiple field and greenhouse experiments should be considered to identify more-efficient PGP inoculants and to determine whether fungi colonize the root and the primary substance responsible for the antioxidant activity for future use in agriculture.

## Figures and Tables

**Table 1:** Identification, plant growth promoting traits and drought stress tolerance of the endophytic and rhizosphere isolates selected for in vitro plant growth-promoting (PGP) traits.

Named	Closest Relative Species	P	I-P	N-A	CAS	EPS	10%D	15%D	25%D	*nifH*	*acdS*
FN1	*Talaromyces pinophilus*	+++	+	ND	++	-	-	-	-	ND	ND
FN2	*Talaromyces purpureogenus*	+++	+	ND	+	-	-	-	-	ND	ND
FN3	*Penicillium* sp.	+++	++	ND	+++	-	-	-	-	ND	ND
FN4	*Talaromyces cellulolyticus*	++	+	ND	-	-	-	-	-	ND	ND
FN5	*Talaromyces purpureogenus*	+	+	ND	-	-	+	-	-	ND	ND
FN11	*Talaromyces flavus*	+++	-	ND	+	-	-	-	-	ND	ND
FN19	*Aspergillus aureolus*	-	-	ND	+	-	-	-	-	ND	ND
FN22	*Chaetomium pilosum*	-	+	ND	++	-	-	-	-	ND	ND
FN24	*Penicillium limosum*	-	-	ND		-	-	-	-	ND	ND
FN30	*Talaromyces purpureogenus*	+++	-	ND	+	-	-	-	-	ND	ND
FNJ6	*Talaromyces purpureogenus*	+++	-	ND	+	-	-	-	-	ND	ND
FNJ7	*Trichocomaceae* sp.	+++	-	ND	+	-	-	-	-	ND	ND
FNJ8	*Hypocreales* sp.	+++	-	-	-	-	√	-	-	ND	ND
N3	*Serratia nematodiphila*	+++	-	+	-	++	1.646	1.225	1.011	√	-
N4	*Pantoea dispersa*	+	-	++	-	+	2.075	1.68	0.898	√	√
N5	*Pantoea ananatis*	+	-	-	+	-	1.524	1.272	0.656	√	√
N6	*Enterobacter* sp.	+	-	-	-	-	1.759	1.379	0.588	√	√
N8	*Citrobacter gillenii*	++	-	++	-	++	1.599	1.19	0.69	-	√
N9	*Acinetobacter* sp.	++	-	++	-	-	1.776	1.523	0.576	√	√
N11	*Klebsiella pneumoniae* subsp.	-	-	++	-	-	1.355	1.012	0.561	√	√
NJ3	*Flavobacterium acidificum*	++	-	-	+	-	0.861	0.639	0.579	√	-
NJ4	*Pseudomonas putida*	+++	-	-	+	-	1.233	0.87	0.853	√	-
NJ5	*Serratia* sp.	+	+	+	-	++	1.135	0.859	0.577	√	-
NJ6	*Citrobacter gillenii*	+	+	+++	-	++	1.264	0.833	0.788	√	-
NJ7	*Klebsiella* sp.	+	+	+	-	+	1.313	0.504	0.45	√	-
NJ10	*Burkholderiaceae bacterium*	++	-	-	-	+	2.109	1.321	0.791	√	√
NJ16	*Serratia marcescens*	-	+	++	-	-	0.943	0.925	0.794	√	-

The list includes the strain taxonomic classification and the results of the physiological tests performed in vitro. **Note:** FNx indicates endophytic fungi; FNJx indicates rhizospheric fungi; Nx indicates different endophytic bacteria; NJx indicates different rhizospheric bacteria; P = organophosphorus medium; I-P = inorganic phosphorus medium; CAS = siderophore production; N-A = N-fixation ability on Ashby medium; EPS = exopolysaccharides release; 10%D, 15%D, and 25%D = 10%, 15%, and 25% polyethylene glycol, indicates the OD_600_ value of bacteria when PEG was mixed to TSB medium.; *nifH* = *nifH* gene; *acdS* = *acdS* gene; ‘+’ indicates different abilities or grades; ‘-’ means negative result in this property; ‘√’ indicates strains have this gene; Blank indicates ND (not determined).

## Supplementary Materials

The following are available online at https://www.mdpi.com/2076-2607/8/9/1329/s1, Figure S1: Representative images of rice inoculated with fungi compared to non-inoculated rice under well-irrigated and drought stress; Figure S2: Representative images of rice inoculated with endophyte and rhizosphere bacteria under well-irrigated and drought stress; Table S1: Identification of the fungal and bacterial isolates; Table S2: Plant growth promotion traits of the cultivable fungi and bacteria isolated from the upland rice roots
