# Supplementary material for: Microbial Diversity of Upland Rice Roots and Their Influence on Rice Growth and Drought Tolerance
Source: Microorganisms. 2020 Aug 31;8(9):1329. doi: 10.3390/microorganisms8091329 (PMC7564600; doi:10.3390/microorganisms8091329)
Supplement: Supplementary file 1 [file microorganisms-08-01329-s001.zip › Supplementary Fig.docx]

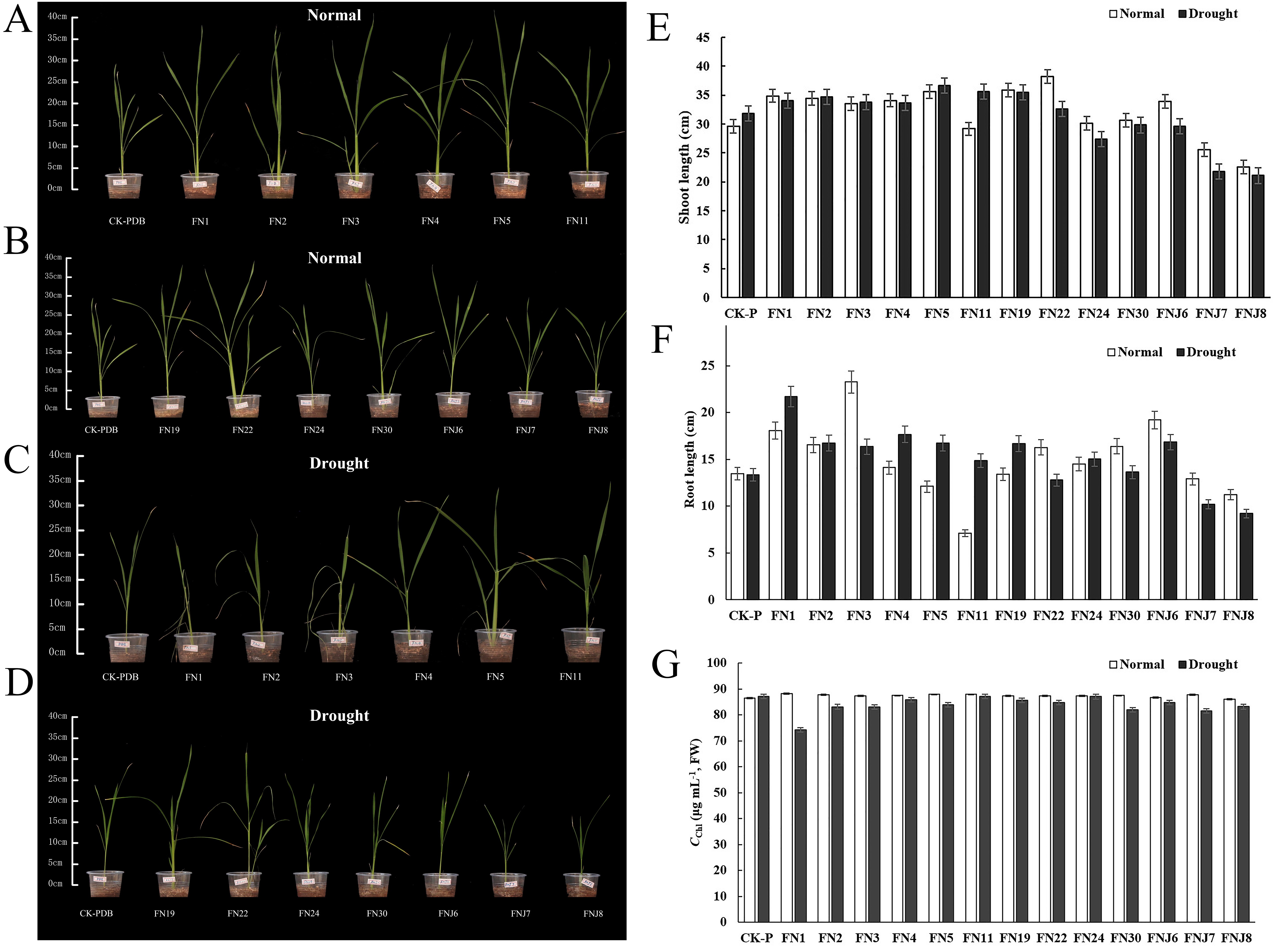


**Supplementary Fig.1** Representative images of differential fungal effects on rice growth and drought tolerance. (A-D) Representative images of rice shoot and root inoculated with endophyte and rhizosphere fungi compared to non-inoculated plants under well-irrigated and drought stress. (A-B) Well-irrigated. (C-D) Drought stress. (E) Shoot length. (F) Root length. (G) Chlorophyll contents. Note: FNx indicates endophytic fungi; FNJx indicates rhizospheric fungi; Nx indicates different endophytic bacteria; NJx indicates different rhizospheric bacteria.


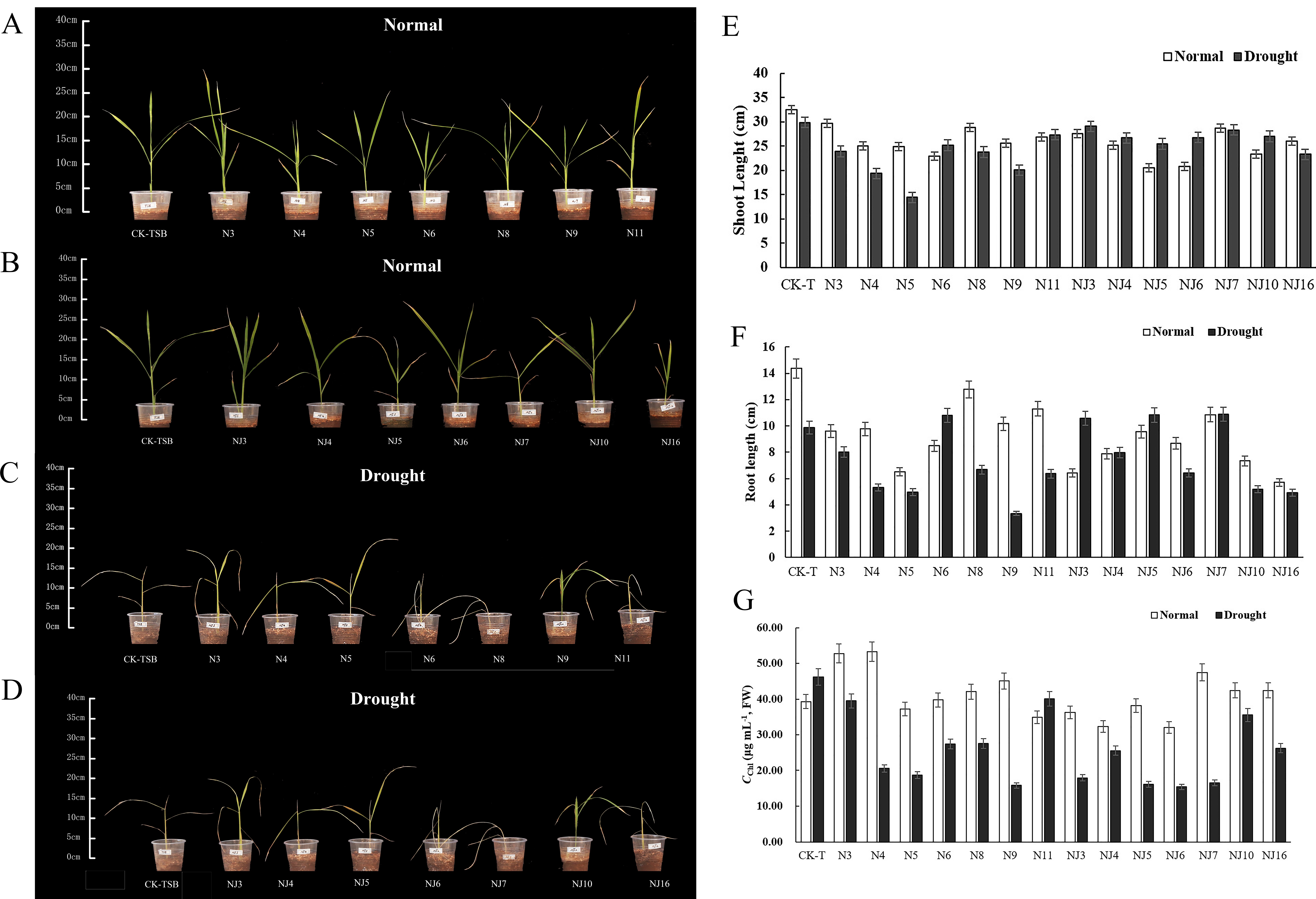


**Supplementary Fig.2** Representative images of differential bacterial effects on rice growth and drought tolerance. (A) Endophyte under well-irrigated. (B) Rhizosphere under well-irrigated. (C) Endophyte under drought stress. (D) Rhizosphere under drought. (E) Shoot length. (F) Root length. (G) Chlorophyll contents.
