# Supplementary material for: Microbial Diversity of Upland Rice Roots and Their Influence on Rice Growth and Drought Tolerance
Source: Microorganisms. 2020 Aug 31;8(9):1329. doi: 10.3390/microorganisms8091329 (PMC7564600; doi:10.3390/microorganisms8091329)
Supplement: Supplementary file 1 [file microorganisms-08-01329-s001.zip › Supplementary Table 1&2.docx]

**Supplementary Table 1. Identification of the fungal and bacterial isolates.** List of the fungal and bacterial strains isolated from upland rice, identified by 16S rRNA and ITS gene sequencing (one representative strain for each ITS group has been sequenced) and blast search in the NCBI, RDP and UNITE public database.

**Supplementary Table 1.1 Identification of the fungal isolates.**

| **NO.** | **Host** | **Nearest match** | **Number** | **Similarity** | **GenBank NO.** |
| --- | --- | --- | --- | --- | --- |
| F1 | PHB1-S1F5(E) | *Talaromyces pinophilus* | 4 | 99% | MF683084.1 |
| F3 | PHB1-S1F5(E) | *Talaromyces purpureogenus* | 2 | 99% | LT558987.1 |
| F4 | PHB1-S1F5(E) | *Penicillium sp.* | 1 | 98% | MK099980.1 |
| F6 | PHB1-S1F5(E) | *Penicillium pinophilum* | 1 | 98% | JQ776546.1 |
| F8 | PHB-XTBG(S0)(E) | *Talaromyces pinophilus* | 7 | 98% | KY999441 |
| F9 | PHB-XTBG(S0)(E) | *Trichocomaceae sp.* | 2 | 99% | JQ999040.1 |
| F17 | AZUCENA(E) | *Talaromyces sp.* | 1 | 98% | MK775999.1 |
| F18 | AZUCENA(E) | *Trichocomaceae sp.* | 1 | 99% | JQ999040.1 |
| F17 | AZUCENA(E) | *Talaromyces pinophilus* | 2 | 99% | KJ621683.1 |
| F21 | AZUCENA(E) | *Neosartorya fischeri* | 2 | 99% | AB674772.2 |
| F23 | PHB1-S1F6(E) | *Talaromyces cellulolyticus* | 1 | 99% | KP996614.1 |
| F24 | PHB1-S1F6(E) | *Talaromyces purpureogenus* | 3 | 99% | MH170875.1 |
| F25 | PHB1-S1F6(E) | *Penicillium pinophilum* | 1 | 99% | JQ776546.1 |
| F27 | PHB1-S1F6(E) | *Talaromyces sp.* | 1 | 99% | MK367754.1 |
| F28 | PHB1-S1F6(E) | *Talaromyces pinophilus* | 2 | 99% | MH170875.1 |
| F30 | PHB1-S1F6(E) | *Talaromyces funiculosus* | 1 | 99% | LN833548.1 |
| F32 | PHB1-S1F8(E) | *Mucor irregularis* | 1 | 97% | MH397499.1 |
| F33 | PHB1-S1F8(E) | *Sarocladium oryzae* | 1 | 98% | MK902763.1 |
| F36 | PHB1-S1F8(E) | *Fusarium sp.* | 1 | 98% | KM655518.1 |
| F37 | PHB1-S1F8(E) | *Talaromyces purpureogenus* | 1 | 99% | LT558987.1 |
| F38 | PHB1-S1F8(E) | *Talaromyces cellulolyticus* | 1 | 97% | KP996614.1 |
| F39 | PHB1-S1F8(E) | *Talaromyces pinophilus* | 2 | 99% | KY999441.1 |
| F40 | PHB1-S1F8(E) | *Pestalotiopsis disseminata* | 1 | 97% | KY999441.1 |
| F43 | Luying46(E) | *Aspergillus aureolus* | 1 | 99% | NG_069978.1 |
| F44 | Luying46(E) | *Thielavia terricola* | 5 | 99% | KY999442.1 |
| F45 | Luying46(E) | *Fusarium solani* | 2 | 98% | MF685335.1 |
| F49 | Luying46(E) | *Neocosmospora rubicola* | 2 | 99% | KM231799.1 |
| F51 | Luying46(E) | *Chaetomium pilosum* | 1 | 99% | NG_069734.1 |
| F52 | Luying46(E) | *Aspergillus udagawae* | 2 | 99% | KY808744.1 |
| F53 | Luying46(E) | *Penicillium limosum* | 1 | 97% | NG_069001.1 |
| F57 | Luying46(E) | *Penicillium chrysogenum* | 1 | 97% | KF998385 |
| F58 | Luying46(E) | *Penicillium sp.* | 1 | 100% | KX611014.1 |
| F59 | Mengwanggu(E) | *Thielavia terricola* | 1 | 98% | KY999442.1 |
| F60 | Mengwanggu(E) | *Nectria haematococca* | 1 | 97% | KJ780750.1 |
| F61 | Mengwanggu(E) | *Fusarium sp.* | 1 | 99% | MN080416.1 |
| F62 | Mengwanggu(E) | *Fusarium oxysporum* | 1 | 97% | HQ130708.1 |
| F63 | Mengwanggu(E) | *Trichoderma sp.* | 1 | 99% | KJ562370.1 |
| F64 | Mengwanggu(E) | *Talaromyces purpureogenus* | 1 | 98% | MK870989.1 |
| F66 | Mengwanggu(E) | *Marasmius nigrobrunneus* | 1 | 99% | KJ778754.1 |
| F67 | Shangmenge(E) | *Trichocomaceae sp.* | 2 | 98% | JQ999040.1 |
| F69 | Shangmenge(E) | *Talaromyces pinophilus* | 1 | 97% | KJ621683.1 |
| F70 | Xiaojingu(E) | *Thielavia sp.* | 1 | 99% | KM268653.1 |
| F71 | Xiaojingu(E) | *Talaromyces pinophilus* | 1 | 98% | KJ621683.1 |
| F72 | Xiaojingu(E) | *Penicillium griseofulvum* | 1 | 98% | MK834749.1 |
| F73 | Xiaojingu(E) | *Penicillium citrinum* | 2 | 98% | KX664347 |
| F74 | Xiaojingu(E) | *Penicillium sp.* | 2 | 99% | HQ699034.1 |
| F76 | Xiaojingu(E) | *Aspergillus aureolus* | 1 | 98% | KY808743.1 |
| FJ2 | PHB1-S1F5(R) | *Talaromyces purpureogenus* | 1 | 97% | MN121629.1 |
| FJ3 | PHB-XTBG(S0)(R) | *Talaromyces pinophilus* | 2 | 96% | CP017345.1 |
| FJ5 | PHB1-S1F8(R) | *Mucor irregularis* | 1 | 97% | MH102381.1 |
| FJ6 | Luying46(R) | *Talaromyces pinophilus* | 5 | 99% | KJ621683.1 |
| FJ7 | Luying46(R) | *Talaromyces purpureogenus* | 3 | 97% | MH170875.1 |
| FJ8 | Luying46(R) | *Acremonium cellulolyticus* | 1 | 90% | AB474749.2 |
| FJ9 | Luying46(R) | *Talaromyces cellulolyticus* | 1 | 99% | KP996614.1 |
| FJ16 | Mengwanggu(R) | *Talaromyces pinophilus* | 5 | 99% | KJ621683.1 |
| FJ17 | Mengwanggu(R) | *Neosartorya sp.* | 1 | 97% | HQ731622.1 |
| FJ19 | Mengwanggu(R) | *Talaromyces purpureogenus* | 7 | 97% | MG986765.1 |
| FJ26 | Mengwanggu(R) | *Trichoderma paraviridescens* | 1 | 97% | MF782827.1 |
| FJ30 | Mengwanggu(R) | Trichoderma hamatum | 1 | 99% | KY225666 |
| FJ31 | Mengwanggu(R) | *Trichoderma sp.* | 1 | 99% | MH550506.1 |
| FJ32 | Mengwanggu(R) | *Thielavia sp.* | 1 | 99% | KM268653.1 |
| FJ33 | Shangmenge(R) | *Talaromyces funiculosus* | 1 | 97% | LN833548.1 |
| FJ34 | Shangmenge(R) | *Talaromyces pinophilus* | 1 | 99% | LC406460.1 |
| FJ39 | Xiaojingu(R) | *Penicillium sp.* | 2 | 97% | MK099980.1 |
| FJ39 | Xiaojingu(R) | *Talaromyces sp.* | 1 | 98% | MK808539.1 |
| FJ40 | Xiaojingu(R) | *Emmia lacerata* | 1 | 99% | MH734799.1 |
| FJ41 | Xiaojingu(R) | *Ceriporia lacerata* | 1 | 98% | KJ780757.1 |
| FJ42 | Xiaojingu(R) | *Talaromyces cellulolyticus* | 1 | 97% | KM458798.1 |
| FJ43 | Xiaojingu(R) | *Aspergillus udagawae* | 4 | 99% | KY808744.1 |
| FJ44 | Xiaojingu(R) | *Talaromyces pinophilus* | 3 | 97% | MF683084.1 |
| FJ45 | Xiaojingu(R) | *Talaromyces purpureogenus* | 2 | 99% | MH170875.1 |

**Supplementary Table1. 2 Identification of the bacterial isolates.**

| **NO.** | **Host** | **Nearest match** | **Number** | **Similarity** | **GenBank NO.** |
| --- | --- | --- | --- | --- | --- |
| 1 | Luying46(E) | *Bacillus qingshengii* | 3 | 98% | NR_133978.1 |
| 6 | Luying46(E) | *Escherichia fergusonii* | 1 | 98% | NR_074902.1 |
| 7 | Luying46(E) | *Klebsiella aerogenes* | 5 | 98% | NR_102499.2 |
| 8 | Luying46(E) | *Bacillus zhangzhouensis* | 3 | 99% | NR_148786.1 |
| 15 | Luying46(E) | *Acinetobacter calcoaceticus* | 1 | 99% | NR_119113.1 |
| 16 | Luying46(E) | *Acinetobacter sp.* | 1 | 98% | KF999999.1 |
| 17 | Luying46(E) | *Acinetobacter lactucae* | 3 | 99% | NR_152004.1 |
| 21 | Mengwanggu(E) | *Klebsiella aerogenes* | 10 | 99% | NR_102499.2 |
| 22 | Mengwanggu(E) | *Siccibacter colletis* | 3 | 98% | NR_134807.1 |
| 23 | Mengwanggu(E) | *Arthrobacter woluwensis* | 1 | 99% | NR_044898.1 |
| 24 | Mengwanggu(E) | *Acinetobacter lactucae* | 2 | 99% | NR_152004.1 |
| 33 | Mengwanggu(E) | *Flavobacterium acidificum* | 12 | 99% | NR_104992.1 |
| 38 | Mengwanggu(E) | *Enterobacter asburiae* | 1 | 99% | MK641844.1 |
| 5P-4 | Mengwanggu(E) | *Serratia nematodiphila* | 1 | 100% | MK447122.1 |
| 5P-5 | Mengwanggu(E) | *Serratia marcescens* | 2 | 99% | NR_114043.1 |
| 52 | 368(E) | *Klebsiella aerogenes* | 12 | 99% | NR_102499.2 |
| 56 | 368(E) | *Stenotrophomonas pavanii* | 1 | 99% | NR_116799.1 |
| 63 | 368(E) | *Erwinia persicina* | 1 | 99% | NR_119964.1 |
| 64 | 368(E) | *Citrobacter gillenii* | 4 | 98% | MF111235.1 |
| 65 | 368(E) | *Klebsiella pneumoniae* | 2 | 98% | KY770799.1 |
| 66 | 368(E) | *Enterobacter cancerogenus* | 1 | 99% | MH714885.1 |
| 67 | 368(E) | *Acinetobacter sp* | 1 | 98% | KP772026.1 |
| 68 | 368(E) | *Enterobacter ludwigii* | 2 | 99% | MH714884.1 |
| 71 | 368(E) | *Enterobacter sp.* | 6 | 99% | MF187599.1 |
| 72 | 368(E) | *Klebsiella pneumoniae subsp.* | 1 | 98% | KP772071.1 |
| 83 | 368(E) | *Acinetobacter lactucae* | 1 | 99% | NR_152004.1 |
| 85 | 368(E) | *Serratia marcescens* | 3 | 98% | NR_114043.1 |
| 5P-7 | 368(E) | *Pseudomonas sp.* | 1 | 99% | FJ976091.1 |
| 5P-10 | 368(E) | *Serratia nematodiphila* | 1 | 98% | MK447122.1 |
| 86 | PHB1-S1(E) | *Enterobacter sp.* | 16 | 99% | MF187599.1 |
| 89 | PHB1-S2(E) | *Serratia nematodiphila* | 4 | 98% | MK447122.1 |
| 99 | PHB1-S3(E) | *Citrobacter gillenii* | 5 | 99% | MF111126.1 |
| 98 | PHB1-S4(E) | *Klebsiella pneumoniae* | 2 | 98% | KJ833791.1 |
| 128 | PHB1-S5(E) | *Klebsiella pneumoniae subsp. pneumoniae* | 2 | 98% | KP772071.1 |
| 134 | PHB1-S6(E) | *Serratia sp.* | 1 | 98% | KR58825.1 |
| 101 | PHB-XTBG(SO)(E) | *Enterobacter sp.* | 1 | 98% | MF187599.1 |
| 102 | PHB-XTBG(SO)(E) | *Serratia nematodiphila* | 3 | 98% | MK1447122.1 |
| 103 | PHB-XTBG(SO)(E) | *Klebsiella pneumoniae subsp. pneumoniae* | 2 | 98% | KP772071.1 |
| 105 | PHB-XTBG(SO)(E) | *Citrobacter gillenii* | 2 | 98% | MF111126.1 |
| 107 | PHB-XTBG(SO)(E) | *Klebsiella pneumoniae* | 1 | 99% | KJ833791.1 |
| 109 | PHB-XTBG(SO)(E) | *Serratia sp.* | 1 | 98% | KR058825.1 |
| 111 | AZUCENA(E) | *Klebsiella pneumoniae subsp. pneumoniae* | 3 | 99% | KP772071.1 |
| 112 | AZUCENA(E) | *Serratia nematodiphila* | 3 | 98% | MK447122.1 |
| 113 | AZUCENA(E) | *Citrobacter gillenii* | 3 | 99% | MF111235.1 |
| 118 | AZUCENA(E) | *Klebsiella pneumoniae* | 1 | 98% | KY770799.1 |
| 5P-1 | AZUCENA(E) | *Pseudomonas sp.* | 1 | 99% | FJ976091.1 |
| 136 | PHB1-S1-F8(E) | *Serratia marcescens* | 3 | 97% | FJ263670.1 |
| 137 | PHB1-S1-F8(E) | *Citrobacter gillenii* | 2 | 98% | MF111235.1 |
| 138 | PHB1-S1-F8(E) | *Uncultured bacterium clone* | 1 | 98% | KX998481.1 |
| 139 | PHB1-S1-F8(E) | *Enterobacter cancerogenus* | 1 | 99% | MH714885.1 |
| 140 | PHB1-S1-F8(E) | *Serratia nematodiphila* | 3 | 99% | MK1447122.1 |
| 142 | PHB1-S1-F8(E) | *Enterobacter sp.* | 4 | 98% | MF187599.1 |
| 145 | PHB1-S1-F8(E) | *Klebsiella pneumoniae subsp. pneumoniae* | 2 | 99% | KP772071.1 |
| 149 | PHB1-S1-F8(E) | *Erwinia toletana* | 1 | 99% | MF111244.1 |
| 151 | 366(E) | *Enterobacter sp.* | 4 | 98% | MF111540.1 |
| 152 | 366(E) | *Klebsiella pneumoniae* | 4 | 98% | KJ833791.1 |
| 153 | 366(E) | *Serratia nematodiphila* | 2 | 98% | MK47122.1 |
| 155 | 366(E) | *Bacillus cereus* | 2 | 99% | MF111577.1 |
| 5P-11 | PHB-XTBG(S0)(E) | *Serratia nematodiphila* | 8 | 99% | MK447122.1 |
| 5P-14 | PHB-XTBG(S0)(E) | *Enterobacter sp.* | 2 | 99% | MF187599.1 |
| J1 | Luying46(R) | *Bacillus qingshengii* | 7 | 92% | NR_133978.1 |
| J2 | Luying46(R) | *Bacillus wiedmannii* | 1 | 98% | NR_152692.1 |
| J3 | Luying46(R) | *Bacillus funiculus* | 1 | 98% | NR_028624.1 |
| J6 | Luying46(R) | *Klebsiella aerogenes* | 12 | 99% | NR_102499.2 |
| J9 | Luying46(R) | *Bacillus dakarensis* | 1 | 99% | NR_147382.1 |
| J14 | Luying46(R) | *Salmonella enterica subsp.* | 1 | 99% | NR_119108.1 |
| J18 | Luying46(R) | *Bacillus alkalitelluris* | 1 | 99% | NR_043210.1 |
| J19 | Luying46(R) | *Pseudomonas mucidolens* | 1 | 99% | NR_114225.1 |
| 5PJ-23 | Luying46(R) | *Serratia nematodiphila* | 2 | 98% | MK447122.1 |
| J25 | Mengwanggu(R) | *Bacillus qingshengii* | 10 | 98% | NR_133978.1 |
| J26 | Mengwanggu(R) | *Klebsiella aerogenes* | 10 | 99% | NR_102499.2 |
| J27 | Mengwanggu(R) | *Acinetobacter lactucae* | 5 | 99% | NR_152004.1 |
| J28 | Mengwanggu(R) | *Bacillus zhangzhouensis* | 1 | 99% | NR_148786.1 |
| J35 | Mengwanggu(R) | *Salmonella enterica subsp.* | 3 | 99% | NR_119108.1 |
| J37 | Mengwanggu(R) | *Sporolactobacillus spathodeae* | 2 | 99% | NR_134816.1 |
| J38 | Mengwanggu(R) | *Cronobacter sakazakii* | 1 | 99% | NR_118449.1 |
| J39 | Mengwanggu(R) | *Burkholderia territorii* | 2 | 98% | NR_136499.1 |
| J44 | Mengwanggu(R) | *Bacillus solisilvae* | 1 | 98% | NR_159143.1 |
| J45 | Mengwanggu(R) | *Bacillus massiliosenegalensis* | 1 | 99% | NR_125590.1 |
| J48 | Mengwanggu(R) | *Erwinia iniecta* | 1 | 99% | NR_137333.1 |
| J57 | Mengwanggu(R) | *Serratia marcescens* | 3 | 98% | NR_114043.1 |
| J198 | Mengwanggu(R) | *Psychrobacillus lasiicapitis* | 1 | 99% | NR_159144.1 |
| 5PJ-25 | Mengwanggu(R) | *Serratia nematodiphila* | 2 | 99% | MF111126.1 |
| J59 | 368(R) | *Bacillus qingshengii* | 1 | 91% | NR_133978.1 |
| J60 | 368(R) | *Acinetobacter lactucae* | 3 | 98% | NR_152004.1 |
| J61 | 368(R) | *Klebsiella aerogenes* | 4 | 99% | NR_102499.2 |
| J63 | 368(R) | *Serratia marcescens* | 1 | 99% | NR_114043.1 |
| J68 | PHB1-S1(R) | *Acinetobacter lactucae* | 1 | 98% | NR_152004.1 |
| J69 | PHB1-S1(R) | *Bacillus qingshengii* | 16 | 92% | NR_133978.1 |
| J70 | PHB1-S1(R) | *Klebsiella aerogenes* | 5 | 99% | NR_102499.2 |
| J71 | PHB1-S1(R) | *Bacillus wiedmanni* | 1 | 98% | NR_152692.1 |
| J77 | PHB1-S1(R) | *Serratia marcescens* | 9 | 99% | NR_114043.1 |
| J83 | PHB1-S1(R) | *Bacillus velezensis* | 2 | 91% | NR_075005.2 |
| 5PJ-1 | PHB1-S1(R) | *Pantoea agglomerans* | 1 | 99% | NR_104992.1 |
| 5PJ-2 | PHB1-S1(R) | *Serratia nematodiphila* | 5 | 99% | NR_114043.1 |
| 5PJ-17 | PHB1-S1(R) | *Serratia sp.* | 1 | 99% | MK447122.1 |
| 5PJ-34 | PHB-XTBG(S0)(R) | *Serratia marcescens* | 7 | 97% | MF111244.1 |
| 5PJ-35 | PHB-XTBG(S0)(R) | *Serratia nematodiphila* | 3 | 97% | MF111999.1 |
| J87 | PHB-XTBG(S0)(R) | *Bacillus qingshengii* | 13 | 99% | NR_133978.1 |
| J90 | PHB-XTBG(S0)(R) | *Bacillus wiedmannii* | 4 | 99% | NR_152692.1 |
| J106 | PHB-XTBG(S0)(R) | *Flavobacterium acidificum* | 1 | 92% | NR_104992.1 |
| J108 | PHB-XTBG(S0)(R) | *Klebsiella aerogenes* | 1 | 98% | NR_102499.2 |
| J111 | AZUCENA(R) | *Acinetobacter lactucae* | 2 | 99% | NR_152004.1 |
| J112 | AZUCENA(R) | *Bacillus qingshengii* | 12 | 99% | NR_133978.1 |
| J115 | AZUCENA(R) | *Serratia marcescens* | 4 | 98% | NR_114043.1 |
| J122 | AZUCENA(R) | *Bacillus manliponensis* | 2 | 98% | NR_125530.1 |
| J123 | AZUCENA(R) | *Bacillus velezensis* | 1 | 92% | NR_075005.2 |
| 5PJ-8 | AZUCENA(R) | *Flavobacterium acidificum* | 1 | 99% | KX580766.1 |
| 5PJ-10 | AZUCENA(R) | *Exiguobacterium sp.* | 1 | 99% | KR058825.1 |
| 5PJ-12 | AZUCENA(R) | *Serratia nematodiphila* | 3 | 99% | MK447122.1 |
| 5PJ-15 | AZUCENA(R) | *Pseudomonas putida* | 1 | 97% | KR109905.1 |
| 5PJ-19 | PHB1-S1-F8(R) | *Serratia nematodiphila* | 3 | 98% | MK447122.1 |
| 5PJ-22 | PHB1-S1-F8(R) | *Exiguobacterium sp.* | 1 | 97% | MK447122.1 |
| J148 | PHB1-S1-F8(R) | *Serratia marcescens* | 2 | 99% | NR_114043.1 |
| J149 | PHB1-S1-F8(R) | *Bacillus qingshengii* | 7 | 99% | NR_133978.1 |
| J153 | PHB1-S1-F8(R) | *Bacillus wiedmannii* | 5 | 99% | NR_152692.1 |
| J156 | PHB1-S1-F8(R) | *Anaerobacillus macyae* | 1 | 99% | NR_025650.1 |
| J160 | PHB1-S1-F8(R) | *Flavobacterium acidificum* | 2 | 98% | NR_104992.1 |
| J165 | PHB1-S1-F8(R) | *Cupriavidus oxalaticus* | 1 | 99% | NR_025018.2 |
| J167 | PHB1-S1-F8(R) | *Klebsiella aerogenes* | 1 | 99% | NR_102499.2 |
| J168 | 366(R) | *Bacillus qingshengii* | 15 | 98% | NR_133978.1 |
| J174 | 366(R) | *Klebsiella aerogenes* | 2 | 98% | NR_102499.2 |
| J179 | 366(R) | *Serratia marcescens* | 3 | 99% | NR_114043.1 |
| J188 | 366(R) | *Bacillus subterraneus* | 1 | 99% | NR_104749.1 |
| 5PJ-28 | 366(R) | *Exiguobacterium sp.* | 1 | 98% | JX035929.1 |
| 5PJ-29 | 366(R) | *Serratia nematodiphila* | 2 | 99% | FJ263679.1 |
| 5PJ-31 | 366(R) | *Enterobacter asburiae* | 1 | 97% | MF111421.1 |
| 5PJ-32 | 368(R) | *Citrobacter gillenii* | 1 | 97% | FJ263679.1 |
| 5PJ-33 | 368(R) | *Klebsiella sp.* | 1 | 98% | MF111540.1 |
| J189 | 368(R) | *Bacillus qingshengii* | 2 | 98% | NR_133978.1 |
| J191 | 368(R) | *Serratia marcescens* | 1 | 99% | NR_114043.1 |
| J200 | 368(R) | *Klebsiella aerogenes* | 1 | 99% | NR_114043.1 |

**Supplementary Table 2. Plant growth promotion traits of the cultivable fungi and bacteria isolated from the upland rice roots (E, R)**. The table includes the results of the screening performed to characterize the *in vitro* direct and indirect PGP potential of the fungal and bacterial strains. P=organophosphorus medium; I-P=inorganic phosphorus medium; CAS=siderophore production; N-A=N-fixation ability on Ashby medium; EPS=exopolysaccharides release; 10%D, 15%D and 25%D= 10%, 15% and 50% polyethylene glycol, indicates the OD600 value of bacteria when PEG was mixed to TSB medium.; ‘+’ indicates different abilities or grades; ‘-’ means not involved.

**Supplementary Table2. 1 Plant growth promotion traits of the cultivable fungi isolated from the root of upland rice (E, R)**.

| **Strain** | **P** | **I-P** | **CAS** | **10%PEG** |
| --- | --- | --- | --- | --- |
| F1 | +++ | + |  |  |
| F3 | +++ | + | √ |  |
| F4 | +++ | ++ | √ |  |
| F6 | ++ | + | √ |  |
| F6 | + | + |  |  |
| F9 |  |  |  | √ |
| F17 |  |  | √ | √ |
| F18 |  |  | √ |  |
| F19 |  |  |  |  |
| F21 |  |  |  |  |
| F24 | +++ |  | √ |  |
| F26 |  |  | √ | √ |
| F27 |  |  | √ | √ |
| F30 | ++ |  | √ |  |
| F32 |  |  | √ | √ |
| F41 |  |  |  | √ |
| F38 | ++ |  |  |  |
| F40 |  |  | √ |  |
| F43 |  |  |  |  |
| F44 |  |  |  |  |
| F45 |  |  | √ |  |
| F52 |  |  | √ |  |
| F53 |  |  |  |  |
| F57 |  |  |  |  |
| F58 |  |  |  |  |
| F60 |  |  |  |  |
| F61 |  |  |  |  |
| F62 | + |  | √ | √ |
| F63 |  |  |  | √ |
| F64 | +++ |  | √ |  |
| F70 |  |  | √ | √ |
| F72 | ++ |  | √ |  |
| F73 |  |  |  |  |
| F74 |  |  |  | √ |
| FJ1 | + |  |  | √ |
| FJ5 |  |  |  |  |
| FJ6 | ++ |  | √ |  |
| FJ8 | ++ |  | √ |  |
| FJ10 | + |  | √ |  |
| FJ14 | +++ |  | √ |  |
| FJ15 | +++ |  | √ |  |
| FJ26 | +++ |  |  | √ |
| FJ29 |  |  |  |  |
| FJ30 | ++ |  |  | √ |
| FJ31 |  |  |  | √ |
| FJ33 |  |  | √ | √ |
| FJ39 |  |  |  |  |

**Supplementary Table2. 2 Plant growth promotion traits of the cultivable bacteria isolated from the root of upland rice (E, R)**.

| **Strain** | **P** | **I-P** | **N-A** | ***acdS*** | **ADF OD** | ***nifH*** | **10%PEG** | **15% PEG** | **25% PEG** |
| --- | --- | --- | --- | --- | --- | --- | --- | --- | --- |
| 5P-2 | ++ |  |  |  | 1.140 | √ | 1.399 | 1.092 | 0.541 |
| 5P-4 | + |  |  |  | 1.026 | √ | 1.253 | 0.976 | 0.549 |
| 5P-13 | +++ |  | + |  | 1.299 | √ | 0.646 | 1.225 | 1.011 |
| ACD33 | +++ |  | ++ | √ | 0.668 | √ | 2.075 | 1.68 | 0.898 |
| ACD36 | +++ |  |  | √ | 0.863 | √ | 1.524 | 1.272 | 0.656 |
| ACD41 | ++++ |  |  | √ | 0.781 | √ | 1.759 | 1.379 | 0.588 |
| ACD53 |  |  |  | √ | 0.584 | √ | 1.381 | 1.179 | 0.613 |
| ACD64 | ++ |  | ++ | √ | 0.858 |  | 1.599 | 1.19 | 0.69 |
| ACD67 | ++ |  | ++ | √ | 0.654 | √ | 1.776 | 1.523 | 0.576 |
| ACD68 | ++ |  |  | √ | 0.699 | √ | 1.487 | 0.985 | 0.571 |
| ACD72 | + |  | ++ | √ | 0.763 | √ | 1.355 | 1.012 | 0.561 |
| 5Pj-2 |  |  |  |  | 1.024 | √ | 1.399 | 1.092 | 0.541 |
| 5Pj-4 |  |  |  |  | 0.974 | √ | 1.253 | 1.092 | 0.549 |
| 5Pj-13 | + |  |  |  | 0.776 | √ | 0.646 | 0.993 | 0.628 |
| 5PJ-3 | + |  |  |  | 1.098 | √ | 1.066 | 0.828 | 0.555 |
| 5PJ-6 | ++ |  |  |  | 1.109 | √ | 0.981 | 0.785 | 0.842 |
| 5PJ-8 | + + |  |  |  | 1.122 | √ | 0.861 | 0.639 | 0.579 |
| 5PJ-15 | +++ |  |  |  | 0.999 | √ | 1.233 | 0.87 | 0.853 |
| 5PJ-17 | + | + | + |  | 0.999 | √ | 1.135 | 0.859 | 0.577 |
| 5PJ-32 | + | + | +++ |  | 1.177 | √ | 1.264 | 0.833 | 0.788 |
| 5PJ-33 | + | + | + |  | 1.173 | √ | 1.313 | 0.504 | 0.45 |
| 5PJ-22-1 |  |  | + |  | 1.020 | √ | 1.024 | 0.542 | 0.777 |
| J106 | + |  |  | √ | 0.988 | √ | 1.736 | 1.249 | 0.683 |
| J165 | + |  |  | √ | 0.999 | √ | 2.109 | 1.321 | 0.791 |
| AJ199 | + |  |  | √ | 0.844 | √ | 1.663 | 1.106 | 0.722 |
| AJ198 | ++ |  |  | √ | 1.073 | √ | 1.508 | 1.033 | 0.645 |
| AJ199 | ++ |  |  | √ | 0.719 | √ | 1.599 | 1.066 | 0.417 |
| AJ197 | + |  |  | √ | 0.651 | √ | 1.246 | 1.115 | 0.535 |
| 5PJ-16 | + | + |  |  | 1.359 | √ | 1.166 | 0.998 | 0.615 |
| 5PJ-18 | ++ | + | ++ |  | 1.070 | √ | 0.843 | 0.925 | 0.798 |
